# Symbiotic performance of grain and wild herbaceous legumes in the Okavango Delta and Tswapong region of Botswana

**DOI:** 10.1007/s13199-017-0515-2

**Published:** 2017-12-04

**Authors:** N. Bernard, M. Losologolo, U. Batlang, S. Ngwako, G. N. Mashungwa, N.M. Tselaesele, F. Pule-Meulenberg

**Affiliations:** Department of Crop Science and Production, Botswana University of Agriculture and Natural Resources, Private Bag 0027, A1 Sebele Content Farm, Gaborone, Botswana

**Keywords:** Biological nitrogen fixation, Legumes, On-farm, Rhizobia, Soil fertility

## Abstract

The low inherent soil fertility, especially nitrogen (N) constrains arable agriculture in Botswana. Nitrogen is usually added to soil through inorganic fertilizer application. In this study, biological nitrogen fixation by legumes is explored as an alternative source of N. The objectives of this study were to measure levels of N_2_ fixation by grain legumes such as cowpea, Bambara groundnut and groundnut in farmers’ fields as well as to estimated N_2_ fixation by indigenous herbaceous legumes growing in the Okavango Delta. Four flowering plants per species were sampled from the panhandle part of the Okavango Delta and Tswapong area. Nitrogen fixation was measured using the ^15^N stable isotope natural abundance technique. The δ^15^N values of indigenous herbaceous legumes indicated that they fixed N_2_ (−1.88 to +1.35 ‰) with the lowest value measured in *Chamaecrista absus* growing in Ngarange (Okavango Delta). The δ^15^N values of grain legumes growing on farmers’ fields ranging from −1.2 ‰ to +3.3 ‰ indicated that they were fixing N_2_. For grain legumes growing at most farms, %Ndfa were above 50% indicating that they largely depended on symbiotic fixation for their N nutrition. With optimal planting density, Bambara groundnuts on farmers’ fields could potentially fix over 90 kg N/ha in some parts of Tswapong area and about 60 kg N/ha in areas around the Okavango Delta. Results from this study have shown that herbaceous indigenous legumes and cultivated legumes play an important role in the cycling of N in the soil. It has also been shown that biological N_2_ on farmer’s field could potentially supply the much needed N for the legumes and the subsequent cereal crops if plant densities are optimized with the potential to increase food security and mitigate climate change.

## Introduction

Apart from low soil moisture, one of the major constraints to increasing arable production in Botswana is the low inherent fertility of soils (Pule-Meulenberg and Batisani 2003). Nitrogen (N) is the most commonly deficient mineral nutrient in soils, often contributing to reduced plant growth and crop yields. It can be supplied to agricultural crops as inorganic fertiliser. However, chemical fertilizers are expensive for the resource poor subsistence farmers, and their production and excessive usage may pollute the environment through greenhouse gas production. Biological N_2_ fixation involving a symbiotic relationship between some members of Rhizobiaceae (“rhizobia”) and the Leguminosae is another source of N for natural and agricultural ecosystems. This legume-rhizobia symbiotic relationship leads to formation of nodules which are sites for nitrogen fixation, where gaseous atmospheric N_2_ is converted into ammonia. The NH_3_ formed is released to the host-plant cells, making it possible for legumes to survive well under low nitrogen soils and provides residual N to the crops following legume plantation in a crop rotation. As shown by Dakora and Keya (1997), up to 10% of the fixed nitrogen can be leaked to the non-legume during the growing season under intercropping.

Legumes that fix N_2_ include domesticated and wild ones. The amounts of N fixed by grain, herbaceous and tree/shrub legumes growing in different parts of Africa indicate a great variability among species, as well as between sampling sites and countries for the same species (Table 1; Andrews et al. 2011). Various researchers in Africa estimated symbiotic fixation in grain legumes such as cowpea, Bambara groundnut, soybean and groundnut using various techniques (Dakora 1985; Dakora et al. 1987; Sanginga et al. 2001; Ncube et al. 2007; Pule-Meulenberg et al. 2010). These grain legumes have been found to contribute significant amounts of N-fixed ranging from 47 kg N/ha by Bambara groundnut in Zimbabwe (Ncube et al. 2007) to 201 kg N/ha by cowpea in Ghana (Dakora et al. 1987). All of the above-mentioned studies were done on-station. It is clear that on-farm results are different, for example, Naab et al. (2009) measured 4.1–34.2 kg N/ha in some villages in the Upper West Region of Ghana, while Pule-Meulenberg et al. (2010) obtained about 156 kg N/ha on-station in the same region of Ghana. Whether on-farm or on-station, research data on symbiotic fixation in Botswana is scarce. However, studies have shown that cowpea growing on farmers’ fields in various parts of Botswana depended on N_2_ fixation for their N nutrition with Ndfa values ranging from 12.5–91.7% (Pule-Meulenberg and Dakora 2009). Thus, actual amounts of N fixed by cowpea on Botswana soils under farm conditions are not known.

**Table 1:** Amount of N_2_ fixed by different legumes from various countries

Legume species	Country	N-fixed (kg/ha)	Reference
Food legumes			
*Phaseolus vulgaris*	Kenya	17–57	Ssali and Keya (1984)
*Vigna unguiculata*	Kenya	24–39	Ssali and Keya (1984)
	Ghana	201	Dakora et al. (1987)
	Zimbabwe	73–79	Mapfumo and Giller (2001)
*Arachis hypogea*	Ghana	32–134	Dakora (1985)
	Nigeria	11–63	Sanginga (2003)
	Kenya	8	Gathumbi et al. (2002)
	Zimbabwe	46–50	Chikowo et al. (2003)
*Glycine max*	Nigeria	94	Sanginga et al. (2001)
	Zimbabwe	115–127	Ncube et al. (2007)
*Cajanus cajan*	Kenya	142	Gathumbi et al. (2002)
*Vigna subterranea*	Ghana	115–127	Ncube et al. (2007)
	Zimbabwe	142	Gathumbi et al. (2002)
Herbaceous Legumes			
*Lablab purpureus*	Nigeria	215	Sanginga et al. (2001)
	Ivory coast	7–70	Nezomba et al. (2008)
*Crotalaria palliada*	Zimbabwe	173	Nezomba et al. (2008)
*Crotalaria ochroleuca*	Zimbabwe	26	Nezomba et al. (2008)
*Crotalaria juncea*	Zimbabwe	58	Nezomba et al. (2008)
*Eriosema elliticum*	Zimbabwe	7	Nezomba et al. (2008)
*Chamaecrista rotundifolia*	Nigeria	144	Sanginga (2003)
*Chamaecrista mimsoides*	Zimbabwe	79	Nezomba et al. (2008)
*Indigofera astragalina*	Zimbabwe	1.5	Nezomba et al. (2008)
*Indigofera errecta*	Zimbabwe	0.5	Nezomba et al. (2008)
*Indigofera astragalina*	Zimbabwe	1.5	Nezomba et al. (2008)
*Tephrosia villosa*	Ivory coast	27–119	Nezomba et al. (2008)
*Tephrosia radicans*	Zimbabwe	1.4	Nezomba et al. (2008)
*Zornia glabra*	Malawi	16	Cadisch et al. (1989)
Tree/shrub legumes			
*Sesbania sesban*	Senegal	43–102	Ndoye and Dreyfus (1988)
	Kenya	52	Gathumbi et al. (2002)
*Leucaena leucocephala*	Tanzania	110	Högberg and Kvarnström (1982)
	Nigeria	304	Danso et al. (1992)
*Senegalia senegal*	Nigeria	< 20	Sanginga et al. (2001)
	Senegal	5.25	Sprent and Parsons (2000)
*Vachellia tortilis*	Senegal	6.24	Sprent and Parsons (2000)
*Faidherbia albida*	Nigeria	< 20	Sanginga et al. (2001)

Besides grain legumes, many herbaceous and tree legumes also form a symbiotic relationship with rhizobia. Herbaceous plants can be used as green manure, where they add the much needed N into soil for the benefit of a subsequent cereal crop. Amounts of N fixed vary according to the legume species. For example, in Zimbabwe, *Crotalaria palliada* fixed 173 kg N/ha (Nezomba et al. 2008) while *Chamaecrista rotundifolia* fixed 144 kg N/ha (Sanginga 2003). Andrews et al. (2011) collated information on symbiotic fixation traits of legumes in natural ecosystems. Their study has shown that indeed dependence on N_2_ fixation (%Ndfa) and the input of N were very varied among the different ecosystems and plant species. For instance, *Pterocarpus lucens* in Senegal exhibited %Ndfa of about 35% while the N input into the semi-arid ecosystem of Senegal was 13–29 kg N/ha/year (also see Sylla et al. 2002). Although some parts of Botswana are endowed with indigenous herbaceous legumes, information on whether they fix N_2_ symbiotically with bacteria as well as their level of dependency on symbiotic fixation for their N nutrition is not known. Pule-Meulenberg and Dakora (2009) established that some tree legumes of the genus *Vachellia* (formerly *Acacia*) depended on symbiotic fixation with Ndfa values ranging from 44 to 94%.

Thus so far, very few studies have measured N fixed under African farm conditions (Adu-Gyamfi et al. 2007; Adjei-Nsiah et al. 2008; Naab et al. 2009; Pule-Meulenberg et al. 2010). Other studies have been done in Australia (Peoples et al. 1995a; Herridge et al. 2005) and other parts of the world under very different conditions to those on African farms. Also, to our knowledge, no study has assessed the level of N_2_ fixation on herbaceous wild leguminous flora of Botswana as well as their dependency on symbiotic fixation. Therefore, the objective of this study were to: i) assess levels of N_2_ fixation on farmers’ fields by grain legumes; ii) measure levels of N_2_ fixation by wild herbaceous legumes and assess their dependency on symbiotic fixation.

## Methodology

### Description of study sites

Field surveys were conducted in the Okavango panhandle and Tswapong during summer season of 2014 when both herbaceous wild and cultivated legumes were at the peak of flowering. In the Okavango panhandle, the survey was conducted at Seronga (18° 50̍ 11̎ S; 22° 18 06 E), Ngarange (18 24 31 S; 22 01 25 E), and Xakao (18 18 17 S; 21 53 16 E), while in Tswapong, this was conducted in Lekobeng (22 44 16 S; 27 11 17 E) and Mmoo-Kokonye (22 45 50 S; 27 12 02 E); farming areas for the village of Lecheng. The two regions were selected because of contrasting physiography. The Okavango panhandle was characterized by higher annual rainfall (471–548). Historically, the soils of the Okavango Delta area were formed from Aeolian wind Kalahari deposits while those of Tswapong area have been weathered in situ from the parent rocks. Tswapong, is characterized by lower annual rainfall (362–397), and the main landforms are the Tswapong Hills and their foot slopes (Pars et al. 1995). Consequently the soils in the eastern part such as those in Lekobeng are slightly more fertile with slightly higher pH value, higher cation exchange capacity (CEC) and P content compared to Xakao soil which is very sandy, has a lower pH, lower CEC and P content (Table 2). The N content of the soil was not determined. However, most parts of Botswana are known to have soils with very low organic matter, and consequently low N (Pule-Meulenberg and Batisani 2003; Pule-Meulenberg and Dakora 2007). The soil texture for the two locations is classified as loamy sand for Lekobeng and as sand for Xakao, both soils having a high sand percentage (Table 2) with low retention of nutrients as implied by the low %clay.

**Table 2:** Properties of soil samples from different study sites

Location	pH (CaCl_2_)	CEC (Cmol/kg)	P (mg/kg)	%Sand	%Silt	%Clay	Soil textural class
Tswapong (Lekobeng)	5.52	5.07	7.93	86.0	3.0	11.0	Loamy sand
Okavango (Xakao)	5.06	1.55	5.13	95.0	2.0	3.0	Sand

#### Plant sampling and processing

In the Okavango panhandle, the survey was conducted at Seronga, Ngarange and Xakao. In Tswapong area, plants were sampled from Lekobeng, a farming area near Lecheng village. At each farm, for every crop (for example, cowpea, Bambara groundnut and groundnut), an area of 20 × 20 m was demarcated where each grain legume was growing and the number of plants counted to estimate the planting density. At each site, four plants per crop species were randomly selected during flowering and dug out. The four plants were collected in separate paper bags and brought back to the laboratory where the roots were gently washed under a stream of water. Plant shoots were separated from roots and oven dried at 60 °C for 48 h and milled to a fine powder (0.5 mm mesh).

At both regions, (Okavango Delta and Tswapong), in addition of sampling in farmers’ fields, a survey of wild herbaceous legumes was carried out around the locations where grain legumes were sampled. At the Okavango Delta, the wild herbaceous legumes sampled belonged to five tribes of the Subfamily Papilionoideae namely; Crotalarieae, Phaseoleae, Milletieae, Indigofereae and Dalbergieae and one tribe of Caesalpinioideae being Cassieae. Whole plants were sampled during their flowering stage. They were dug out and further treated in the same manner as grain legumes. Plants were identified in the field using books and where there doubts or where they were not in the books, samples were pressed and brought to the Botswana University of Agriculture and Natural Resources (BUAN) Herbarium or the Botswana National Herbarium and Botanic Gardens in Gaborone for identification. Some reference herbaceous plants (non N_2_ fixing) such as weeds growing in close proximity to the sampled legumes were also sampled to estimate soil N taken up by the legume.

#### Determination of δ ^15^N and % Ndfa

The natural abudance of ^15^N/^14^N and %N of field grain legumes from Xakao in Okavango Delta and Lekobeng in Tswapong, and of herbaceous legumes from the Okavango Delta and reference plants were determined by a Thermo Finnigan Delta Plus XP stable light isotope mass spectrometer (Fixon Instrument SPA, Strada Rivolla, Italy) coupled via a Conflo III device to Thermo1112 Flash elemental analyser. About 2.0 mg of each pulverized sample was weighed into a tin capsule (Elementary Microanalysis LTD, Okehampton, UK) and run against two internal reference plant materials namely *Nasturtium sp.* and *Vachellia sp*. The internal standards had been calibrated against an IAEA standard for N, which is atmospheric air. The isotopic composition of ^15^N was measured as the difference in the number of atoms of ^15^N to ^14^N in atmospheric N_2_ according to Junk and Svec (1958) and Mariotti (1983) as follows:

$$ {\updelta}^{15}\mathrm{N}\ \left(\raisebox{1ex}{$0$}\!\left/ \!\raisebox{-1ex}{$00$}\right.\right)=\frac{\left({}^{15}\mathrm{N}{/}^{14}\mathrm{N}\right)\mathrm{sample}\hbox{-} \left({}^{15}\mathrm{N}{/}^{14}\mathrm{N}\right)\mathrm{standard}}{\left({}^{15}\mathrm{N}{/}^{14}\mathrm{N}\right)\mathrm{standard}}\ast 1000 $$

The proportion of N derived from the atmosphere, (%Ndfa) was calculated according to Shearer and Kohl (1986) as follows:

$$ \% Ndfa=\left[\frac{\left({\updelta}^{15}\mathrm{Nref}\right)\hbox{-} {\updelta}^{15}\mathrm{Nleg}\Big)}{\left({\updelta}^{15}\mathrm{Nref}\hbox{-} \mathrm{Bvalue}\right)}\right]\mathrm{x}100 $$

Where; δ^15^Nref is the mean ^15^N natural abundance of a non-N_2_-fixing reference plants, δ^15^Nleg is the mean ^15^N natural abundance of the legume (shoot) and the B value is the ^15^N natural abundance of legume shoots which were totally dependent on biological N_2_ fixation for their N nutrition. The B values used in this study were sought from literature as shown in Tables 3 and 4. For some of the studied legumes, there exist other values in literature. For example, Howieson and Dilworth (2016) have collated B values of some grain and pasture legumes; −1.08, −0.88 and −1.61‰ for *Crotalaria* sp., *Arachis hypogea* and *Vigna unguiculata* respectively. Values for *Crotalaria* sp. and *Vigna unguiculata* are similar to the ones used in this study while for *Arachis hypogea* the B value used in our study is substantially different in magnitude from that of Howieson and Dilworth (2016), probably due to genetic differences.

**Table 3:** Herbaceous legumes plants found at the Okavango Delta and Tswapong

Okavango Delta	Tswapong
*Indigofera flavicans*	*Zornia glochidiata*
*Indigofera tinctoria*	*Chamaecrista biensis*
*Chamaecrista biensis*	*Vigna unguiculata* subsp. *dekindtiana*
*Indigofera astragalina*	*Chamaecrista absus*
*Indigofera daleoides*	*Tephrosia purpurea*
*Tephrosia purpurea*	*Rhyncosia totta*
*Tephrosia lupinifolia*	
*Chamaecrista absus*	
*Crotalaria astragalus*	
*Crotalaria sphaerocarpa*	
*Crotalaria pisicarpa*	
*Vigna unguiculata* subsp. *dekindtiana*	
*Rhyncosia totta*	
*Zornia glochidiata*

**Table 4:** B values of varied plant species obtained from literature

Plant	B value (‰)	Reference
*Arachis hypogea*	−1.4	Okito et al. (2004)
*Indigofera* sp	−1.5	Okito et al. (2004)
*Chamaecrista absus*	−1.7	de Freitas et al. (2012)
*Crotalaria* sp	*−*1.1	Gathumbi et al. (2002)
*Vigna subterranea*	−1.4	Nyemba and Dakora (2010)
*Vigna unguiculata* subsp	−1.7	Okito et al. (2004)
*Vigna unguiculata*	−1.8	Pule-Meulenberg and Dakora (2009)
*Tephrosia purpurea*	−2.0	Raddad et al. (2005)

The amount of N-fixed was calculated as Maskey et al. (2001):

$$ \mathrm{N}-\mathrm{fixed}=\left( Ndfa/100\right)x\  legume shoot\ N $$

#### Statistical analysis

Statistical analyses were carried out with the Statistical Analysis Software (SAS 9.4) (SAS Institute, Carey, NC). Data on δ ^15^N, % Ndfa, N-fixed and N content were subjected to analysis of variance (ANOVA) after testing for normality. Where means were statistically different they were separated using the Fisher’s Least Significant Difference (LSD) method at 5% significant level.

## Results

### Mean δ^15^N values of reference plants

In addition to herbaceous legume species, non N_2_ fixing plant species were also sampled as reference plants for the calculation of %Ndfa using natural abundance technique. These included *Ocimum americanum*, *Panicum* sp., an unknown grass species, *Erlangea musera*, *Cassia italica*, *Termnalia sericea* and *Senna obtusfolia* (Table 5). Mean δ^15^N values of reference plants ranged from 4.02–5.99‰.

**Table 5:** Reference plants and their δ^15^N (‰) values

Plant	δ^15^N (‰)
*Ocimum americunum*	5.99
*Panicum sp*	5.39
*Unknown grass species*	5.47
*Erlangea musera*	4.02
*Cassia italica*	4.79
*Terminalia sericea*	4.05
*Senna obtusfolia*	4.64

### Occurrence of herbaceous of legumes in the two study sites

Table 3 presents the results of the legume survey that was undertaken in the Okavango Delta and the Tswapong region. In both cases the survey was conducted at four locations. Table 3 shows that more herbaceous legume plant species were found in the higher rainfall Okavango Delta compared to Tswapong area. Further, the two sites appear to have different soil chemical properties, with Tswapong presenting higher pH, CEC and phosphorus (Table 2).

### A comparison of plant growth, δ ^15^N values, %Ndfa and N-fixed of grain legumes in farmers’ fields in Tswapong and Okavango Delta

At flowering, there were significant differences in the dry matter of grain legumes growing at the various sites where sampling was done (Table 6). There were no differences in the dry matter accumulated by groundnuts growing in Lekobeng (L), Xakao (Ngando) (XN) and Xakao (Motenya) (XM). Regarding Bambara groundnut, plants growing in XN were significantly smaller than those grown in Lekobeng and XM. The symbiotic performance of the grain legumes differed significantly among sites at the Okavango Delta and Tswapong area (Table 6). Bambara groundnut growing in Lekobeng exhibited the lowest δ ^15^N value, followed by groundnut from Xakao. In fact, groundnut had the lowest overall δ ^15^N value irrespective of sampling site. For example at Ngando’s farm in Xakao, at Motenya’s farm in Xakao and in Lekobeng, δ^15^N values of groundnuts were 0.0, 0.5 and 0.5 ‰ respectively. It is noteworthy that Bambara groundnut showed the most enriched δ^15^N values in Okavango Delta region regardless of the farm where they were growing (Table 6). Interestingly, the most depleted value of δ^15^N was measured in Bambara groundnut (−1.2‰) in Lekobeng. Cowpea was only sampled from Okavango in Xakao at Ngando farm and its δ ^15^N value was not significantly different from that of Bambara groundnut growing in the same farm (Table 6).

**Table 6:** Shoot biomass and nitrogen fixation characteristics of various grain legumes sampled from farmers’ fields in Tswapong and Okavango regions of Botswana

Agro ecological zones	Crop	Biomass g.plant^−1^	δ^15^N (‰)	%Ndfa	N content (mg.plant^−1^)	N-fixed (mg plant^−1^)	Plants/0.4 ha	N fixed (kg.ha^−1^)	Potential N fixed (kg.ha^1^)
Okavango	*Vigna unguiculata XN*	5.4 ± 0.2c	1.9 ± 0.5b	46.0 ± 6.6c	187.8 ± 6.5c	85.9 ± 12.2 cd	82	0.2 ± 0.9c	12.9 ± 1.8d
Tswapong	*Arachis hypogea* L	31.3 ± 6.1a	0.5 ± 0.3c	69.3 ± 4.1b	654.7 ± 155.3ab	440.0 ± 84.6b	75	0.8 ± 0.2bc	40.8 ± 7.3c
Okavango	*Arachis hypogea* XN	28.9 ± 1.8a	0.5 ± 0.3c	71.0 ± 4.5b	838.8 ± 62.2a	594.6 ± 55.3ab	78	1.2 ± 0.2ab	59.5 ± 5.5bc
Okavango	*Arachis hypogea* XM	30.5 ± 4.9a	0.0 ± 0.4d	78.7 ± 5.4b	797.9 ± 130.9a	618.0 ± 96.5a	72	1.1 ± 7.0ab	61.8 ± 7.5b
Okavango	*Vigna Subterranea* XN	7.6 ± 1.3c	1.8 ± 0.3b	50.3 ± 4.4c	211.0 ± 33.5c	110.1 ± 25.9c	78	0.3 ± 2.1c	21.2 ± 5.6d
Tswapong	*Vigna Subterranea L*	25.7 ± 2.9ab	−1.2 ± 0.2e	96.4 ± 2.7a	651.6 ± 75.2ab	624.9 ± 66.3a	85	1.3 ± 0.1a	93.7 ± 9.9a
Okavango	*Vigna Subterranea* XM	15.4 ± 4.7b	3.3 ± 0.4a	28.8 ± 5.8d	431.3 ± 130.0bc	110.6 ± 33.9c	92	0.2 ± 2.6c	16.6 ± 4.0d
	F-statistics	8.76***	20.60***	21.57***	6.97***	17.90***		19.08***	20.07***

Means ± SE in a column with dissimilar letters are significantly different. * = *p* < 0.05, ** = *p* < 0.01, *** = *p* < 0.001; XN = Xakao (Ngando), Xakao (Motenya), L = Lekobeng

All the grain legumes sampled were dependent on symbiotic fixation for their N nutrition. For instance, because Bambara groundnut growing in Lekobeng had the most depleted δ ^15^N value and it was the most highly dependent on N_2_ fixation with an Ndfa of 96.4%. Interestingly, of all the sampled grain legumes, the least dependent on N_2_ fixation was also Bambara groundnuts in Xakao at Motenya farm with Ndfa of 28.8%. Data revealed that regardless of sampling site, groundnut generally depended on symbiotic fixation for its N nutrition with Ndfa values greater than 70%.

There were significant differences in amounts of N fixed among grain legumes grown at various sites (Table 6). Cowpea and Bambara groundnut grown in Xakao at Ngando (XN) farm fixed the least amount of N, 85.9 and 110.6 mg N/plant respectively while Bambara grown in Xakao at Motenya (XM) farm, groundnut grown at Motenya (XM) farm and groundnut grown in Xakao at Ngando (XN) farm fixed the highest amounts N of 624.9, 618.0 and 594 mg N/plant respectively. When translated to hectare basis using the planting density used by farmers, N-fixed amounts for Bambara grown at XN, groundnut grown at XM and XN become 1.3, 1.1 and 1.2 kg N/ha respectively. The relationship between shoot biomass of grain legumes is shown in Fig. 2; it shows that N-fixed amounts increased with increasing biomass.

### A comparison of plant growth, δ ^15^N values, %Ndfa and N-fixed of wild indigenous herbaceous legumes from sites at Okavango Delta

**Fig. 1:**
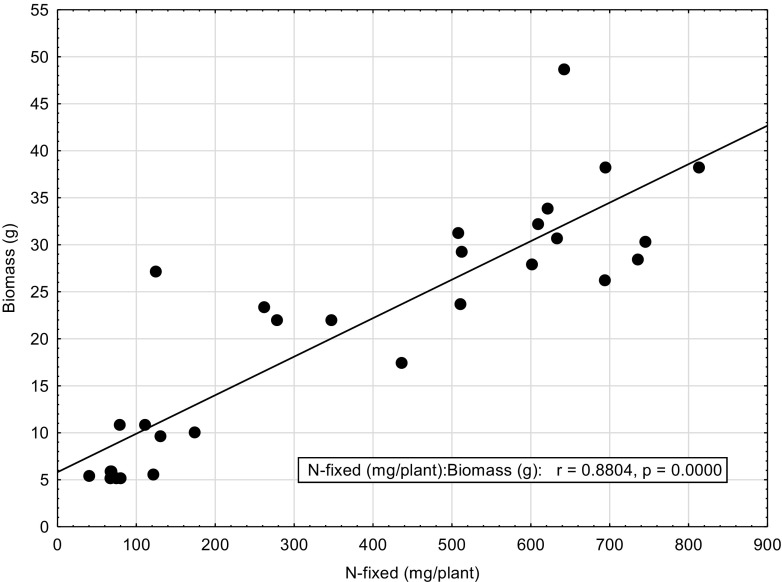
The relationship between shoot biomass and amount of N_2_ fixed by grain legumes

**Fig. 2:**
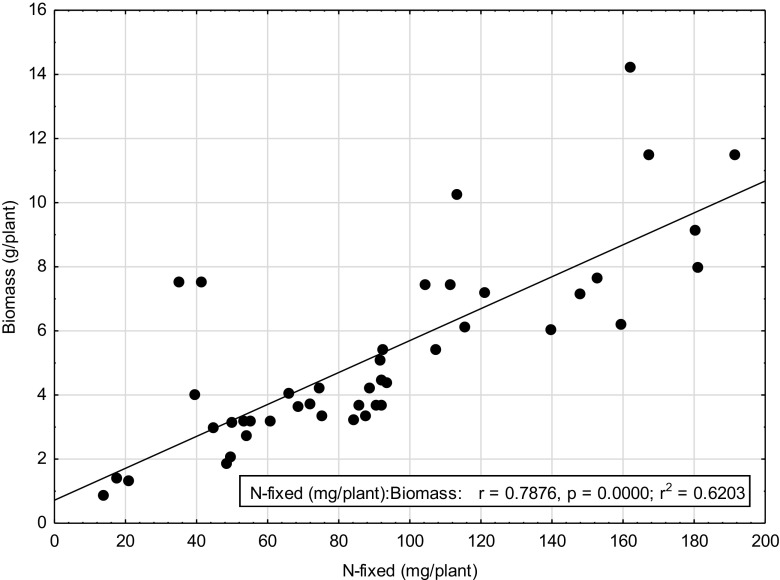
The relationship between plant biomass and amount of N_2_ fixed by wild legumes

Because the various legumes were different and therefore had varying growth patterns, there were significant differences in their shoot dry matter at flowering stage (Table 7). There were significant differences in the symbiotic efficacy of the herbaceous legumes at the various sites of the Okavango Delta. Most legumes exhibited considerably depleted values of δ^15^N as shown by mostly negative figures. Plant species from Ngarange exhibited the most depleted values of δ^15^N (‰) while plant species from Xakao were comparatively enriched (Table 7). Consequently, the legumes showed a high dependency on symbiotic fixation, values ranging between 57.5% by *Indigofera flavicans* sampled from Xakao to 100% by *Chamaecrista absus* from Ngarange. The N contents were significantly different among species with *Indigofera daeloides* from Xakao showing the highest amount of N per plant and *Crotalaria astragalus* the least. All the sampled legumes from the Okavango Delta depended on symbiotic N fixation with %Ndfa values of more than 50% (Table 7). Because % Ndfa were high, amounts of N-fixed (mg/plant) were in some cases very close to the total N content. For example in *Tephrosia* sp., the total N content was 147.8 mg/plant while N-fixed was 143.4 mg/plant (Table 7) indicating a strong dependence of symbiotic fixation. The shoot biomass of wild herbaceous legumes was correlated with the amount of N-fixed per plant (*r* = 0.8804; *p* = 0.000), that is, N-fixed amounts increased with increasing biomass (Figs. 1 and 2).

**Table 7:** Shoot biomass and nitrogen fixation characteristics of various wild legumes collected from the Okavango Delta

Location	Tribe	Plant name	Biomass g/plant	δ^15^N (‰)	%Ndfa	N content (mg.plant^−1^)	N-fixed (mg.plant^−1^)
Ngarange	Cassieae	*Chamaecrista absus*	7.1 ± 0.8abc	−1.9 ± 0.1 g	100.0 ± 1.4a	134.8 ± 16.4abc	134.8 ± 17.0ab
Ngarange	Phaseoleae	*Vigna unguiculata* subsp. *dekindtiana*	2.6 ± 0.7e	−0.8 ± 0.2d	84.5 ± 3.7de	72.6 ± 15.8 cd	68.1 ± 15.7c
Ngarange	Crotalarieae	*Crotalaria* sp	3.7 ± 0.3de	−0.6 ± 0.2d	81.6 ± 3.4de	99.1 ± 6.5bc	80.8 ± 5.4c
Ngarange	Milletieae	*Tephrosia* sp	6.2 ± 0.7bcd	−1.5 ± 0.1f	97.1 ± 1.6ab	147.8 ± 19.1ab	143.4 ± 18.6a
Ngarange	Indigoferae	*Indigofera* sp	4.1 ± 1.0de	−1.1 ± 0.2e	89.3 ± 2.8bc	87.7 ± 18.6c	78.2 ± 16.0c
Seronga	Crotalarieae	*Crotalaria sphaerocarpa*	4.4 ± 0.9cde	−0.7 ± 0.2d	91.0 ± 2.4bc	97.5 ± 24.0bc	88.0 ± 21.2bc
Seronga	Crotalarieae	*Crotalaria astragalus*	2.5 ± 0.7e	0.2 ± 0.2b	76.6 ± 3.5d	50.1 ± 15.1d	39.5 ± 13.2c
Xakao	Phaseoleae	*Vigna unguiculata* subsp. *dekindtiana*	2.4 ± 0.6e	1.3 ± 0.5a	56.4 ± 7.8e	81.1 ± 20.5bcd	46.0 ± 12.0c
Xakao	Milletieae	*Tephrosia lupinifolia*	5.4 ± 1.2 cde	0.6 ± 0.1b	64.1 ± 0.9de	124.9 ± 28.9abc	79.3 ± 17.7c
Xakao	Indigoferae	*Indigofera daleoides*	8.4 ± 1.8a	0.1 ± 0.2c	77.1 ± 2.2d	174.1 ± 29.1a	135.7 ± 25.7ab
Xakao	Indigoferae	*Indigofera flavicans*	9.9 ± 1.6a	1.4 ± 0.1a	57.5 ± 2.0e	148.8 ± 48.4ab	87.9 ± 30.4bc
		F-Statistics	5.78***	23.82***	20.87***	2.44*	3.70**

Means ± SE in a column with dissimilar letters are significantly different. * = p < 0.05, ** = p < 0.01, *** = p < 0.001

## Discussion

The herbaceous legume survey conducted in some villages of the Okavango Delta and in Tswapong region revealed a larger number of plant species in the Okavango Delta compared to the Tswapong region. While 14 plant species were recorded in the Delta, only seven were found in Tswapong (Table 2), attributed to the higher mean annual rainfall, about 600 mm in the Okavango, and between 397 and 431 mm in Tswapong area. Earlier studies showed that the number of putative N_2_ fixing tree legumes of the genus *Vachellia* decreased with increasing aridity (Pule-Meulenberg and Dakora 2009), where the wetter Ngwaketse region had more trees than the drier Kgalagadi, suggesting that soil moisture was a major constraint to N_2_ fixation in tree legumes under prevailing environmental conditions of Botswana.

Nitrogen fixation data in Botswana are very scarce. In other countries, N_2_ fixation information from experimental stations are freely available (Ncube et al. 2007, Adjei-Nsiah et al. 2008, Peoples et al. 1995, Pule-Meulenberg et al. 2010). In the African continent, on farm N_2_ fixation data is very limited (Naab et al. 2009; Pule-Meulenberg and Dakora 2009). But, without studies on levels of symbiotic fixation under farm conditions, it is not possible to optimize the contribution of nitrogen by grain legumes on African soils. In this study, symbiotic traits of grain legumes were measured on plants grown on farmers’ fields in the Okavango Delta in the northern part of Botswana and in Tswapong area in the east using the ^15^N natural abundance technique. Results showed that for some grain legumes (e.g. groundnuts), N_2_ fixation varied among villages and between the two regions (Table 6). Values of δ^15^N also showed that Bambara groundnut fixed N_2_ and that the highest fixation among all legumes was measured in Lekobeng in the Tswapong region. However, despite the low δ^15^N values exhibited by grain legumes, and the high dependence on symbiotic fixation, amounts of N-fixed on farmers’ fields were very low due to low planting densities (Table 6). These low N-fixed figures (0.2 to 1.3 kg N/ha) are comparable to those measured by Naab et al. (2009) in the Upper West region of Ghana where their lowest N-fixed was 7.4 kg N/ha, despite a low δ^15^N value of 0.85‰ also attributed to a suboptimal planting density. Recalculating N-fixed using optimal densities of 150,000 plants/ha for cowpea, 100,000 plant/ha for groundnut and 150 00 plant/ha for Bambara groundnut led to potentially much higher values of N-fixed of 12.9 kg N/ha for cowpea, 40.8–61.8 kg N/ha for groundnut and 16.6–93.7 kg N/ha for Bambara groundnut.

In addition to the sampling of grain legumes from farmers’ fields, wild herbaceous legumes were sampled from the Okavango Delta. A comparison of the symbiotic performance of the herbaceous legumes growing in the panhandle part of the Okavango Delta revealed that all the legumes depended on N_2_ fixation for their N nutrition (Tables 6 and 7). In a study that investigated ^15^N signatures of nodulated legumes in the Cerrado and neighbouring regions of Brazil, Sprent et al. (1996) observed that species of *Chamaecrista* exhibited δ^15^N values that indicated that they were fixing atmospheric N_2_. In this study, a herbaceous species of *Chamaecrista absus* equally fixed N_2_ with a δ^15^N value of −1.9‰. All species exhibited depleted δ^15^N values of less than 5‰, indicating that they were fixing N_2_ from the atmosphere implying that they have a significant role in the nitrogen cycle in that area by adding nitrogen to the ecosystem and using the fixed N for supporting their growth. Lemaire et al. (2015) isolated N_2_ fixing symbionts from root nodules of legumes collected from the Core Cape Subregion (CCR) also known as the Fynbos area of the Western Cape region of South Africa. There are some commonalities between Lemaire et al. (2015) and the current study in that their legumes belonged to similar tribes namely Crotalarieae, Phaseoleae, Milletieae, Indigofereae and Dalbergieae. Although they did not measure N_2_ fixation in those plants, they did establish that they were nodulated and formed symbiotic fixation. Data on associated microsymbionts are not presented in this study.

The variation in N_2_ fixation by both wild herbaceous and field legumes collected from various locations can be attributed to the differences in competitive capability and effectiveness of the indigenous rhizobial population at each location as supported by Martins et al. (2003), that under different soils the rhizobial population differs in species composition and symbiotic effectiveness. Also, supporting studies by Mapfumo and Giller (2001) observed that differences in N fixation were mainly due to variability in soil physical, biological and chemical properties for both rhizobia and plants, environmental conditions, cropping history and the management practices together with adaptability of the symbiotic partners to environmental conditions.

In general, when compared to legumes collected from farmer’s fields, wild herbaceous legumes had higher % Ndfa, for example, *Chamaecrista absus* from Ngarange totally depended on symbiotic fixation for its N nutrition (Table 7). Higher % Ndfa shows that the legumes derived majority of N from fixing atmospheric nitrogen, indicating that they were growing under N deficient soils compared to field legumes. Equally higher N fixation by wild legumes due to low soil N status could be enhanced by the co-existence of legumes with non-legumes in natural ecosystems. A supporting study by Cramer et al. (2007) concluded that non legumes were beneficial for up taking and utilizing N in the soil, creating N deficiency in the soil which sequentially boost legumes to biologically fix nitrogen to their optimal level with the consequent improvement of %Ndfa. Generally, δ^15^N of reference plants in this study were rather depleted (Table 5). A study by Spriggs et al. (2003) has shown that one of the reasons for low δ^15^N of non-N_2_-fixing plants could be mycorrhization. Apparently mycorrhizal fungi discriminate against the heavier ^15^N isotope during transfer of N from the fungus to the host plant, leaving the host plant with a more depleted in ^15^N. This implies that dependency on symbiotic fixation could be underestimated, thus N-fixed amounts could be higher than reported.

## Conclusion

In conclusion, grain legumes growing on farmers’ fields in the Okavango Delta and the Tswapong region of Botswana varied in their levels of N_2_ fixation. Regarding δ^15^N signatures, the most depleted value was measured on Bambara groundnut growing in Tswapong region, probably due to low soil N content. Despite the generally low δ^15^N of grain legumes, ranging between −1.9 to 1.4 ‰, and high Ndfa values ranging from 28.8 to 96.4%, amounts of N-fixed were very low due to low planting densities. Using optimal planting densities, grain legumes could potentially fix substantial amounts of nitrogen, for example, about 94 kg N/ha can be fixed by Bambara groundnut in Lekobeng. The wild herbaceous legumes growing in the Okavango Delta equally fixed N_2_, showing high dependence (56–100%) on symbiotic fixation. Taken together, this study has shown that symbiotic N_2_ fixation can play an important role in contributing to the N economy of crops in farmers’ fields. We have also shown that despite a decrease in the diversity of wild herbaceous legumes with increasing aridity, plants are fixing N_2_ and are dependent on biological nitrogen fixation for their N nutrition.
